# Refining the predictive variables in the “Surgical Risk Preoperative Assessment System” (SURPAS): a descriptive analysis

**DOI:** 10.1186/s13037-019-0208-2

**Published:** 2019-08-20

**Authors:** William G. Henderson, Michael R. Bronsert, Karl E. Hammermeister, Anne Lambert-Kerzner, Robert A. Meguid

**Affiliations:** 1 Surgical Outcomes and Applied Research program, Department of Surgery, University of Colorado School of Medicine, Aurora, CO USA; 2 Adult and Child Center for Health Outcomes Research and Delivery Science, University of Colorado School of Medicine, Aurora, CO USA; 3 Department of Biostatistics and Informatics, Colorado School of Public Health, Aurora, CO USA; 4 Division of Cardiology, Department of Medicine, University of Colorado School of Medicine, Aurora, CO USA; 5 VA Eastern Colorado Health Care System, Department of Veterans Affairs Medical Center, Aurora, CO USA; 6 Division of Cardiothoracic Surgery, Department of Surgery, University of Colorado Denver | Anschutz Medical Campus, 12631 E. 17th Avenue, C-310, Aurora, CO 80045 USA

## Abstract

**Background:**

The Surgical Risk Preoperative Assessment System (SURPAS) is a parsimonious set of models providing accurate preoperative prediction of common adverse outcomes for individual patients. However, focus groups with surgeons and patients have developed a list of questions about and recommendations for how to further improve SURPAS’s usability and usefulness. Eight issues were systematically evaluated to improve SURPAS.

**Methods:**

The eight issues were divided into three groups: concerns to be addressed through further analysis of the database; addition of features to the SURPAS tool; and the collection of additional outcomes. Standard multiple logistic regression analysis was performed using the 2005–2015 American College of Surgeons National Surgical Quality Improvement Participant Use File (ACS NSQIP PUF) to refine models: substitution of the preoperative sepsis variable with a procedure-related risk variable; testing of an indicator variable for multiple concurrent procedure codes in complex operations; and addition of outcomes to increase clinical applicability. Automated risk documentation in the electronic health record and a patient handout and supporting documentation were developed. Long term functional outcomes were considered.

**Results:**

Model discrimination and calibration improved when preoperative sepsis was replaced with a procedure-related risk variable. Addition of an indicator variable for multiple concurrent procedures did not significantly improve the models. Models were developed for a revised set of eleven adverse postoperative outcomes that separated bleeding/transfusion from the cardiac outcomes, UTI from the other infection outcomes, and added a predictive model for unplanned readmission. Automated documentation of risk assessment in the electronic health record, visual displays of risk for providers and patients and an “About” section describing the development of the tool were developed and implemented. Long term functional outcomes were considered to be beyond the scope of the current SURPAS tool.

**Conclusion:**

Refinements to SURPAS were successful in improving the accuracy of the models, while reducing manual entry to five of the eight variables. Adding a predictor variable to indicate a complex operation with multiple current procedure codes did not improve the accuracy of the models. We developed graphical displays of risk for providers and patients, including a take-home handout and automated documentation of risk in the electronic health record. These improvements should facilitate easier implementation of SURPAS.

**Electronic supplementary material:**

The online version of this article (10.1186/s13037-019-0208-2) contains supplementary material, which is available to authorized users.

## Background

Clinical risk assessment and decision support tools built into the electronic health record (EHR) are becoming more common. To be successful, these tools need to be user friendly, require minimal data input, apply to broad clinic populations, and integrate seamlessly into clinic work flow [1]. We have been developing a preoperative risk assessment and decision support tool, the Surgical Risk Preoperative Assessment System (SURPAS), for surgical patients at the University of Colorado Hospital that has these desired features and is based on the large database from the American College of Surgeons’ National Surgical Quality Improvement Program Participant Use File (ACS NSQIP PUF). This development has involved an iterative process including initial prediction model development [2–4]; development of a prototype tool integrated into the local EHR; focus groups of patients, surgeons, and administrators to review the prototype [5]; pilot testing; and then refinements based upon the focus groups and pilot testing.

In the initial modeling, we found that the 18 30-day postoperative complications collected in the ACS NSQIP could be statistically grouped into six clusters of complications: infectious; pulmonary; cardiac/bleeding; venous thromboembolic; renal; and neurological [2]. Along with 30-day mortality and overall morbidity, this resulted in eight important postoperative adverse outcomes being predicted by the SURPAS tool. We also found that eight preoperative variables could predict risk of these eight adverse outcomes almost as well as using models including up to 40 preoperative ACS NSQIP variables, across the nine surgical subspecialties represented in the ACS NSQIP database. These eight preoperative variables included four patient factors (age, American Society of Anesthesiology physical status classification (ASA class), functional health status prior to surgery (FHS), and presence of systemic sepsis within 48 h of surgery) and four operative factors (operation complexity as measured by work Relative Value Unit (wRVU), in- or outpatient operation, primary surgeon specialty, and whether or not the operation was an emergency).

In integrating the SURPAS tool into the local EHR (Epic Systems, Verona, WI), we found that only two of the eight SURPAS preoperative predictor variables could be reliably obtained from the EHR at the time of the patient’s preoperative encounter—patient age and specialty of the primary surgeon. The other six predictor variables need to be entered by the surgical team.

The focus groups of patients, surgeons, and administrators expressed a number of concerns and/or recommendations for refinements to the prototype SURPAS tool: [5]

1. Related to the eight preoperative predictor variables, preliminary work in integration of SURPAS into the local EHR suggested that systemic sepsis within 48 h of surgery would be a difficult variable to assess at the preoperative encounter;
2. Also related to the preoperative variables, there was concern about the adequacy of the wRVU of the primary operation accounting for the complexity of the operation, particularly in operations involving multiple Current Procedural Terminology (CPT) codes;
3. Although a factor analysis of the postoperative complications suggested six clusters of complications, some of the complications that were clustered together are likely addressed by different processes of care; therefore, some should be made distinct postoperative adverse outcomes (e.g., cardiac complications and bleeding were separated; surgical site infections and urinary tract infections (UTIs) were separated, etc.);
4. Hospitalizations causing the patient to spend time away from home and family were identified by our patient partners as important patient concerns; therefore, the risk of unplanned re-hospitalization following surgery should be included as an adverse postoperative outcome;
5. The SURPAS tool should provide documentation of the risk information and discussion with the patient and family in the patient’s medical record;
6. The SURPAS tool should provide risk information to the patients and their families in a printed and easily understood format to help them understand and remember details of the informed consent process;
7. The SURPAS tool should provide answers to “frequently asked questions” (FAQs) to facilitate the implementation of the tool and to foster collaborative discussions with patients; and
8. One patient who experienced debilitating depression after his operation suggested incorporating a risk for adverse psychological or cognitive effects postoperatively.

Some of these concerns/recommendations needed to be addressed through further analysis of the ACS NSQIP database (Items #1, 2, 3, 4); some could be addressed by adding features to the SURPAS tool (Items #5, 6, 7); and another required the collection of additional outcomes (Item #8). The purpose of this paper is to report on the further statistical analyses and features added to the SURPAS tool in response to these concerns and recommendations. Our hypotheses were that: (1) Elimination of the preoperative sepsis variable would have negligible effect on fit of the SURPAS predictive models; (2) A predictor variable representing CPT-specific event rate would be an important predictor variable to add to the model in place of preoperative sepsis; (3) A predictor variable indicating multiple CPT codes for complex operations would not significantly add to model fit; and (4) Additional adverse outcomes of unplanned readmission, UTI, and bleeding would be successfully predicted by the refined eight SURPAS predictor variables.

## Methods

### Study design

This study was a retrospective, observational study using the 2005–2015 American College of Surgeons National Surgical Quality Improvement Program Participant Use File (ACS-NSQIP PUF). The database includes a systematic sample of major operations performed at > 700 participating hospitals in nine surgical specialties (general, orthopedic, gynecology, urologic, neurosurgery, otolaryngology, thoracic, plastic, and vascular surgery). Trained clinical nurse reviewers collect preoperative, operative, and 30-day postoperative adverse outcomes on a systematic sample of patients at their hospitals using a standardized protocol and data definitions. The study was ruled exempt from review by the Colorado Multiple Institutional Review Board (COMIRB) because it involved using completely de-identified data from a national database.

### Issue 1

In the previous SURPAS publications, we found that eight preoperative predictor variables could accurately predict eight different postoperative complications across a broad range of surgical operations [2–4]. The final SURPAS models were developed using standard multiple logistic regression analysis, with the adverse postoperative outcomes as the dependent variables and the eight preoperative predictor variables as the independent variables [4]. To address the issue of dropping preoperative sepsis from the group of eight preoperative predictor variables, we performed similar logistic regression models including only seven preoperative variables (eliminating preoperative sepsis) and compared the c-indexes and Brier scores of the seven-variable models to the original eight-variable models.

We added a predictor variable that we call “CPT-specific event rate.” This variable is defined as the event rate of the CPT code of the primary operation for the outcome being modeled, calculated from the ACS NSQIP database for the years 2005–2015 that included over 4.6 million observations from over 700 participating hospitals nationwide. We reasoned that CPT-specific event rate calculated from past operations should be a good predictor of the outcome for future operations of the same type (CPT code). We then compared the c-indexes and Brier scores of the eight-variable models that included “CPT-specific event rate” with the original eight-variable models that included preoperative sepsis.

### Issue 2

To address the issue of operations with multiple CPT codes being more complex than is represented by the wRVU value, we ran nine-variable models adding an indicator variable for operations with multiple CPT codes (0 = no, 1 = yes) and compared the c-indexes and Brier scores for these nine-variable models to the eight-variable models that included CPT-specific event rate to see if performing operations involving multiple CPT codes added to the risk of the patient. We used an indicator variable for multiple CPT codes, rather than using the anticipated number of CPT codes or the actual CPT codes because we reasoned that at the preoperative encounter, the surgeon might know that the operation would involve multiple CPT codes but might not know exactly which ones would be involved or how many.

### Issues 3 & 4

To address the issues related to the complication clusters and hospitalizations we developed models for a new set of eleven adverse postoperative outcomes that separated bleeding/transfusion from the cardiac outcomes, UTI from the other infection outcomes, and unplanned readmission as an additional adverse postoperative outcome. To validate the new eight-variable models, we randomly split the cases 50:50 into developmental and test datasets, developed the models in the developmental dataset and then tested the developed models in the test dataset and compared c-indexes and Brier scores between the developmental and test samples. We hypothesized that CPT-specific event rate would be one of the more important predictor variables of the eight-variable set of predictors. To assess this, we ran forward selection logistic regression models forcing all eight predictor variables into the models, and compared orders of entry of the variables across the eleven adverse outcomes.

### Issue 5

To address the documentation of the risk information and discussion with the patient and family we added a feature to SURPAS that would automatically generate a pre-operative note in the patient’s EHR summarizing the patient’s individualized preoperative risks compared to the national average for patients undergoing the same operation (Fig. 2), and a note that these data were discussed with the patient.

### Issue 6

To address the issue of providing a graphical display of the patient’s risks to the patient and family, we added a feature to SURPAS to allow a pictograph of the individual patient’s risk compared to the national average to be printed out to give to the patient at the preoperative encounter (Fig. 3). We gave the focus groups of patients several different examples of how these data could be graphically displayed (including a bar graph, pictograph with stick figures, pictograph with boxes, pie graph, sparkplug display, clock graph display, and a table of numerical values) [6–8]. The majority of patients preferred the pictograph for the display of the risks [9].

### Issue 7

To address the issue of providing answers to “frequently asked questions” we developed an “About” section for SURPAS to describe the development of the tool and the underlying methodology (Fig. 4).

### Issue 8

We believed that issues such as long term outcomes and risk for adverse psychological or cognitive effects from the operation were beyond the scope of the current SURPAS development. These will likely involve a different type of data collection and collection longitudinally over time, both before and after the operation. But this focus group comment has prompted us to explore the potential for adding patient-reported outcomes to the SURPAS tool, which will be the subject of future grant applications and publications.

## Results

Additional file 1 Figure S1 presents the STROBE diagram for the development of the analytic database. In the period 2005–2015, there were 4.6 million operations in the ACS NSQIP database. We eliminated 0.6% of the operations for not being one of the nine surgical specialties targeted by the ACS NSQIP and SURPAS (general, orthopedic, vascular, gynecology, urology, otolaryngology, plastic, thoracic, and neurosurgery), and 0.8% of the operations for having missing data on key SURPAS predictor variables (functional health status, ASA class, age, emergency operation, or inpatient/outpatient operation). This resulted in 4.54 million operations being included in the analytic file (98.6% of the total operations). Additional file 1: Table S1 presents the patient characteristics, including the SURPAS predictor variables, and the rates for the eleven postoperative adverse outcomes for these 4.54 million patients.

### Issue 1

In Table 1, we see that elimination of the preoperative sepsis predictor variable had very little effect on the c-indexes and Brier scores for predicting the eleven outcome variables. C-indexes were decreased by 0.001 to 0.013 units, or percent decreases of 0.1 to 1.7%, while Brier scores were increased by 0.0000 to 0.0018 units, or percent increases of 0.0 to 3.9%.

**Table 1:** Comparison of Discrimination and Calibration of the Full Eight Variable SURPAS Model with Models without Systemic Sepsis^a^

Adverse outcome	C-index			Brier Score		
	Full Model	Full Model with No Systemic Sepsis	Difference	Full Model	Full Model with No Systemic Sepsis	Difference
30-day mortality	0.926	0.922	−0.004	0.0102	0.0103	0.0001
Morbidity	0.801	0.793	−0.008	0.0904	0.0922	0.0018
Readmission	0.699	0.698	−0.001	0.0488	0.0489	0.0001
Infection	0.774	0.761	−0.013	0.0445	0.0450	0.0005
Bleeding/Transfusion	0.850	0.846	−0.004	0.0419	0.0421	0.0002
Pulmonary	0.890	0.882	−0.008	0.0228	0.0237	0.0009
UTI	0.750	0.749	−0.001	0.0145	0.0145	0.0000
VTE	0.765	0.760	−0.005	0.0086	0.0086	0.0000
Cardiac	0.869	0.866	−0.003	0.0062	0.0062	0.0000
Renal	0.859	0.853	−0.006	0.0059	0.0059	0.0000
Neurologic	0.829	0.828	−0.001	0.0021	0.0021	0.0000

^a^Abbreviations: *SURPAS* Surgical Preoperative Risk Assessment System, *UTI* Urinary tract infection, *VTE* Venous thromboembolism

When CPT event rate was substituted for preoperative sepsis in a new eight-variable model, c- indexes were increased by 0.002 to 0.031 units, or percent increases of 0.2 to 4.0% (Table 2). Brier scores did not change for five of the eleven adverse postoperative outcomes, were reduced for four additional outcomes by 0.0001 to 0.0024 units (percent reductions of − 0.2% to − 2.8%), and were increased for only two outcomes by 0.0001 units (percent increase of 1.0%) and 0.0005 units (percent increase of 2.2%). The second column in Table 2 gives the c-indexes for the SURPAS prediction models that are in current use. The c-index is above 0.90 for one outcome (mortality, 0.928), between 0.80 and 0.89 for seven outcomes (0.893 for pulmonary, 0.875 for bleeding/transfusion, 0.871 for cardiac, 0.863 for renal, 0.840 for stroke, 0.823 for overall morbidity, and 0.805 for infection), and between 0.70 and 0.79 for three outcomes (0.788 for VTE, 0.776 for UTI, and 0.723 for unplanned readmission).

**Table 2:** Comparison of Discrimination and Calibration of the Full Eight Variable SURPAS Model with Models without Systemic Sepsis but with the Addition of CPT Specific Event Rates^a^

Adverse outcome	C-index			Brier Score		
	Full Model	Full Model with CPT Event Rate	Difference	Full Model	Full Model with CPT Event Rate	Difference
30-day mortality	0.926	0.928	0.002	0.0102	0.0103	0.0001
Morbidity	0.801	0.823	0.022	0.0904	0.0880	−0.0024
Readmission	0.699	0.723	0.024	0.0488	0.0487	−0.0001
Infection	0.774	0.805	0.031	0.0445	0.0442	−0.0003
Bleeding/Transfusion	0.850	0.875	0.025	0.0419	0.0408	−0.0011
Pulmonary	0.890	0.893	0.003	0.0228	0.0233	0.0005
UTI	0.750	0.776	0.026	0.0145	0.0145	0.0000
VTE	0.765	0.788	0.023	0.0086	0.0086	0.0000
Cardiac	0.869	0.871	0.002	0.0062	0.0062	0.0000
Renal	0.859	0.863	0.004	0.0059	0.0059	0.0000
Neurologic	0.829	0.840	0.011	0.0021	0.0021	0.0000

^a^Abbreviations: *SURPAS* Surgical Preoperative Risk Assessment System, *UTI* Urinary tract infection, *VTE* Venous thromboembolism

### Issue 2

To examine whether adding an indicator variable for multiple CPT codes would improve the SURPAS prediction models, we compared nine-variable models (with the addition of an indicator variable for multiple CPT codes) to our eight-variable models (Table 3). The addition of the indicator variable minimally increased the c-indexes by 0.000 to 0.002 units (0.0 to 0.2%), and minimally decreased the Brier scores by 0.0000 to 0.0004 units (decreases of 0.0 to 0.5%).

**Table 3:** Comparison of Discrimination and Calibration of the SURPAS without Systemic Sepsis but with the Addition of CPT Specific Event Rates with Models adding an Indicator for Multiple CPTs^a^

Adverse outcome	C-index			Brier Score		
	Full Model with CPT Event Rate	Same Model plus Multiple CPT Indicator	Difference	Full Model with CPT Event Rate	Same Model plus Multiple CPT Indicator	Difference
30-day mortality	0.928	0.929	0.001	0.0103	0.0103	0.0000
Morbidity	0.823	0.824	0.001	0.0880	0.0876	−0.0004
Readmission	0.723	0.723	0.000	0.0487	0.0487	0.0000
Infection	0.805	0.806	0.001	0.0442	0.0441	−0.0001
Bleeding/Transfusion	0.875	0.877	0.002	0.0408	0.0406	−0.0002
Pulmonary	0.893	0.894	0.001	0.0233	0.0232	−0.0001
UTI	0.776	0.777	0.001	0.0145	0.0145	0.0000
VTE	0.788	0.789	0.001	0.0086	0.0086	0.0000
Cardiac	0.871	0.871	0.000	0.0062	0.0062	0.0000
Renal	0.863	0.865	0.002	0.0059	0.0059	0.0000
Neurologic	0.840	0.84	0.000	0.0021	0.0021	0.0000

^a^Abbreviations: *SURPAS* Surgical Preoperative Risk Assessment System, *UTI* Urinary tract infection, *VTE* Venous thromboembolism

### Issues 3 & 4

Models were developed for a revised set of eleven adverse postoperative outcomes that separated bleeding/transfusion from the cardiac outcomes, UTI from the other infection outcomes, and added a predictive model for unplanned readmission. Table 4 presents the internal validation statistics for the eight-variable SURPAS prediction models, not including preoperative sepsis and including CPT-specific event rate, for the eleven SURPAS adverse postoperative outcomes. The table gives the order of entry of the eight SURPAS prediction variables across the eleven different postoperative adverse outcomes and the average order of entry. CPT-specific event rate was the first variable to enter the models for nine of the eleven adverse postoperative outcomes (all but the models for 30-day mortality and cardiac complications in which ASA class was the first variable to enter). Comparing the c-indexes and Brier scores of the prediction models in the developmental and test datasets, only one of the eleven c- indexes (for UTI) showed the expected decline going from the developmental to the test dataset and that decline was only 0.001 unit, and all changes in Brier scores from the development to the test dataset were within 0.002 units, indicating excellent internal validation.

**Table 4:** Order of Entry of Predictor Variables in the Final SURPAS Models and C-indexes and Brier Scores for the Development and Test Datasets in the Internal Validation Study

Characteristics^a^	30-day Mortality	Morbidity	Unplanned Readmission	Respiratory	Infection	UTI	VTE	Cardiac	Bleeding	Renal	Stroke	Average Rank
CPT-specific event rate	2	1	1	1	1	1	1	2	1	1	1	1.2
ASA class	1	2	2	2	3	2	3	1	2	2	2	2.0
Inpatient/outpatient	6	3	3	3	2	3	2	4	3	3	4	3.3
Primary surgeon specialty	7	4	4	6	4	4	6	6	4	4	5	4.9
Age (years)	4	7	7	7	6	5	4	3	5	7	3	5.3
Functional health status	3	5	5	4	7	6	5	7	8	6	6	5.6
Emergency operation	5	6	8	5	5	8	7	5	7	5	7	6.2
Work relative value unit	8	8	6	8	8	7	8	8	6	8	8	7.5
Model c-index	0.928	0.823	0.724	0.893	0.805	0.777	0.788	0.871	0.875	0.863	0.839	
Model Brier score	0.0103	0.0879	0.0485	0.0234	0.0438	0.0145	0.0086	0.0062	0.0406	0.0060	0.0021	
Validation c-index	0.931	0.823	0.724	0.893	0.805	0.776	0.790	0.877	0.876	0.870	0.875	
Validation Brier score	0.0102	0.0880	0.0483	0.0232	0.0437	0.0144	0.0086	0.0063	0.0407	0.0059	0.0020	

^a^Abbreviations: *ASA class* American Society of Anesthesiology physical status classification, *CPT* Current Procedural Terminology, *SURPAS* Surgical Preoperative Risk Assessment System, *UTI* Urinary tract infection, *VTE* Venous thromboembolism

### Issues 5, 6, and 7

**Fig. 1:**
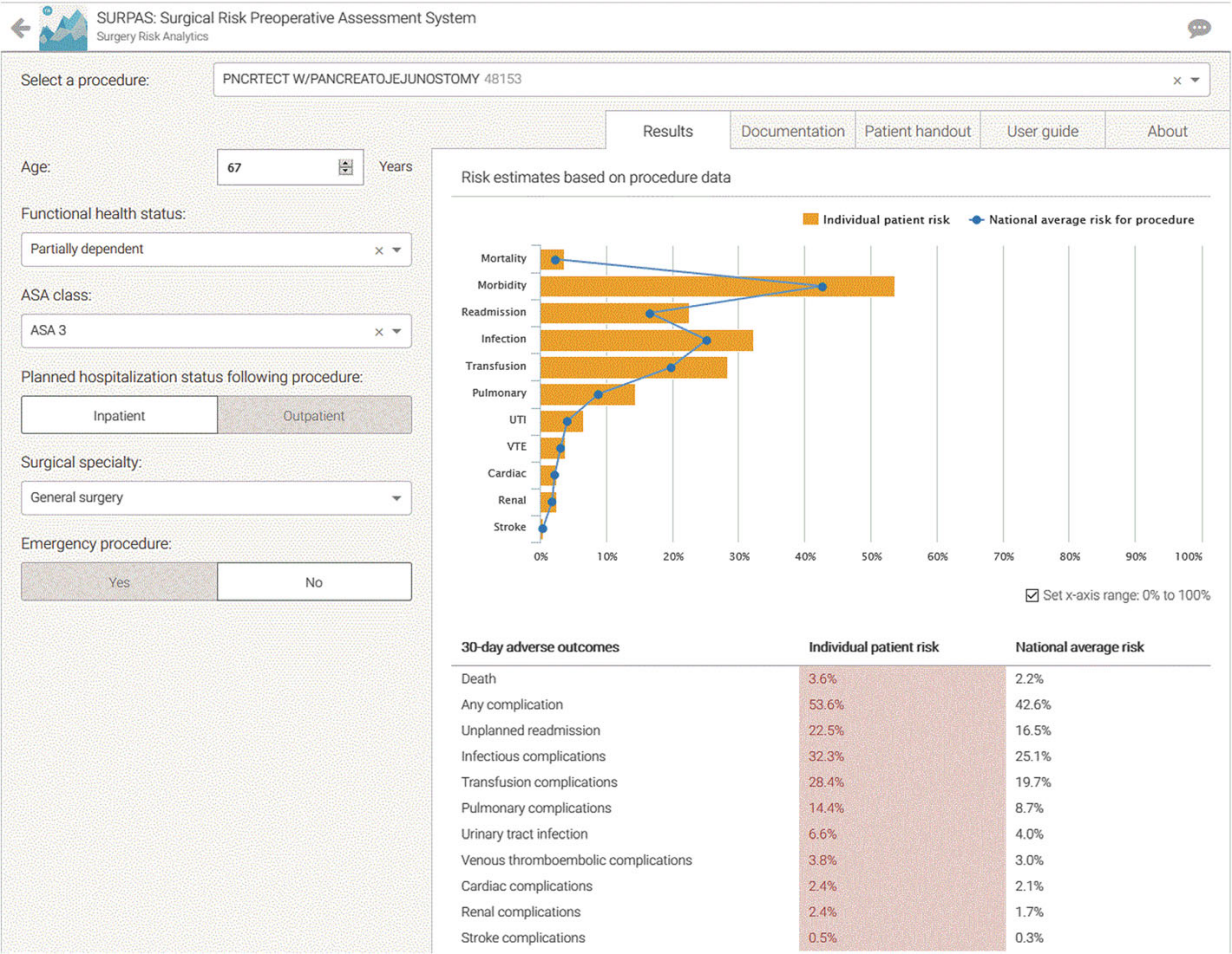
SURPAS Input and Output Screen

**Fig. 2:**
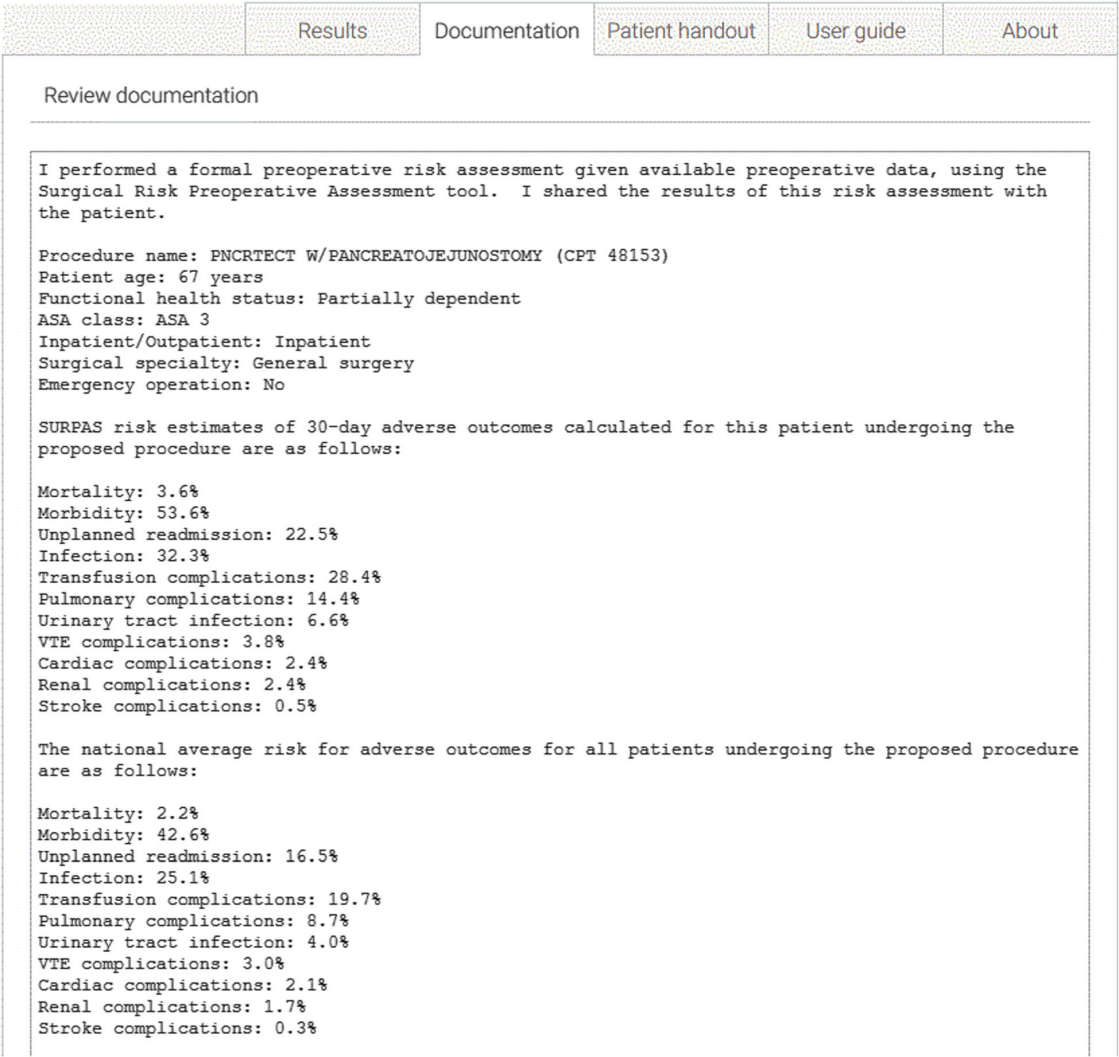
SURPAS Documentation Screen

**Fig. 3:**
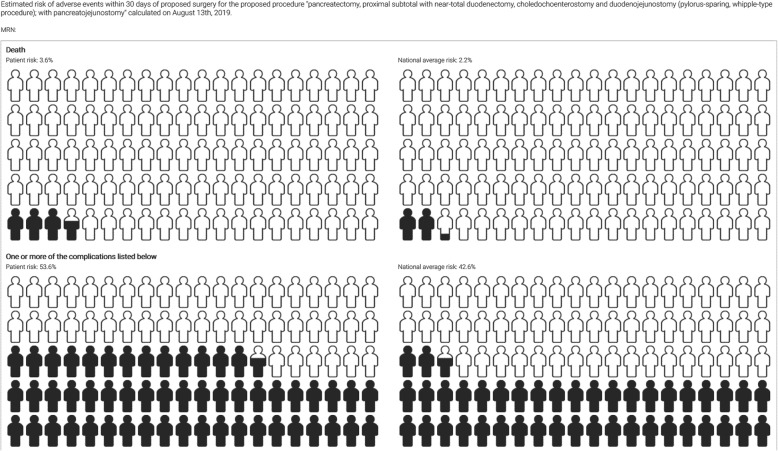
SURPAS Patient handout, abbreviated

**Fig. 4:**
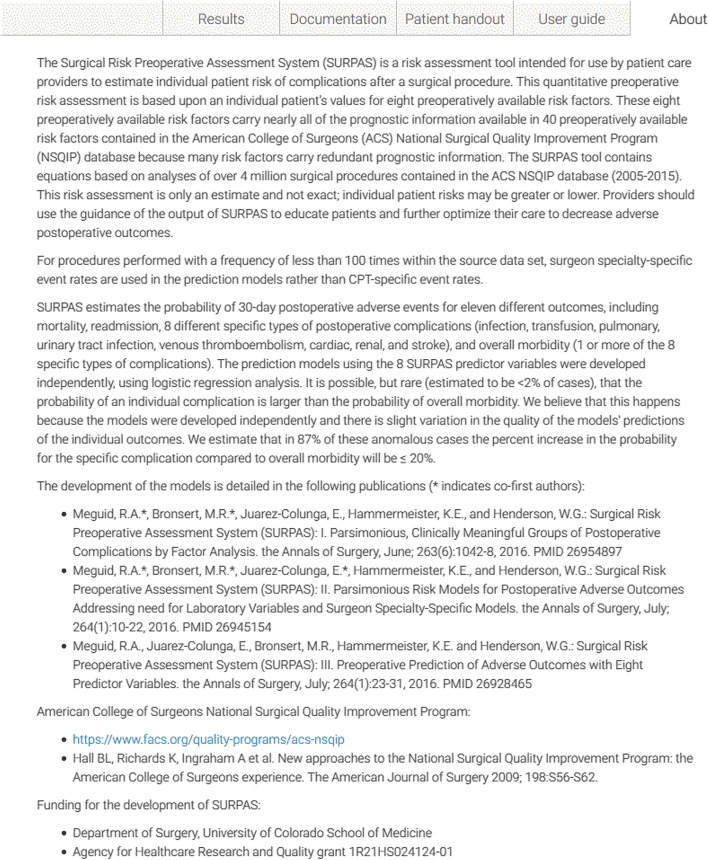
SURPAS About section

Figure 1 shows the SURPAS screen seen by the surgeon at the preoperative visit for a patient undergoing a pancreatojejunostomy. The data input values are on the left: name and/or CPT code of the primary operation; age of the patient (automatically populated from the EHR); functional health status; ASA class; inpatient/outpatient procedure; surgical specialty of the primary surgeon (automatically populated from the EHR); and whether the surgical procedure is done on an emergency basis. Once the CPT code is specified, a table look-up can obtain values for wRVU and CPT-specific event rates. Individualized patient risks for the eleven postoperative adverse outcomes (yellow bars) compared to national averages (blue dots) are given on the right both graphically and in table form. A preoperative note is automatically created for import into the patient’s EHR (Fig. 2). A pictograph of the patient risks compared to national averages (Fig. 3—showing abbreviated pictograph for mortality and overall morbidity) for all eleven of the postoperative adverse outcomes can be printed out for the patient to keep. An “About” section (Fig. 4) can be referenced by the provider to explain the SURPAS tool, and a “User guide” demonstrates to providers how to use the SURPAS tool.

### Issue 8

Long term functional outcomes are beyond the scope of SURPAS due to lack of long term and functional outcomes in the ACS NSQIP PUF. However, these may possibly be available through incorporation of patient reported outcomes such as those measured by the Patient Reported Outcomes Measurement Information System, and piloted by the ACS NSQIP in 2018 (personal communication from Dr. Jason Lyu) [10].

## Discussion

In this paper, we settled on the revised SURPAS prediction models currently in use in the implementation and dissemination of SURPAS at the UCHealth hospitals. Based on feedback from our focus groups of patients, surgeons, and administrators we added three postoperative adverse outcomes to the original eight SURPAS outcomes—bleeding, UTI, and unplanned readmission. We showed that the 11 SURPAS prediction models have good to excellent c-indexes and Brier scores. Preoperative sepsis was replaced as a predictor variable because it was difficult ascertaining this variable preoperatively from the EHR and is unlikely to be present in elective surgical patients, and in its place we added CPT-specific event rate calculated from the large, national ACS NSQIP database of 4.54 million operations. Dropping the preoperative sepsis variable did not have a significant effect on the c-indexes and Brier scores for the prediction models, but adding CPT-specific event rate did significantly improve these measures for several of the prediction models. Another advantage of the CPT-specific event rate is that it does not require additional data input – once the provider enters the name or CPT code of the operation, internal look-ups obtain the values for wRVU and CPT-specific event rates. We reasoned that the simple addition of an indicator for multiple concurrent procedures would be easier for the provider to anticipate, than actually identifying and listing the additional procedures in the SURPAS tool while minimizing intrusion into the compressed preoperative encounter. We showed that adding this did not significantly change the c-indexes and Brier scores for the models, implying that our models do tend to account for the complexity of operations with multiple CPT codes. This might happen if the multiple CPT codes tend to often occur together so that the risk of the complex operation is already built into the CPT code of the primary operation through the CPT-specific event rate. The internal validation study showed that there was very little loss of discrimination and calibration in going from the developmental to the test dataset.

We believe that adding the features of the automatically generated preoperative note, the pictograph of the individual patient risks compared to the national averages, which can be printed out and handed to the patient at the preoperative visit, and the “About” section and “User guide” in the SURPAS tool will enhance the utility of SURPAS to patients and providers. We believe the ability to provide a patient with their risk of postoperative adverse event, review it with them in a meaningful manner, and then provide a hard copy of this information so that they may further consider the risks after leaving the encounter and more clearly relate them to their family will improve their engagement in shared decision making [11].

The mention of cognitive effects of the operation by one patient in the focus groups prompted us to explore the possibility of adding patient reported outcomes to SURPAS. Adverse effects from operations on traits such as cognition, pain, physical functioning, or performance of activities of daily living require longitudinal measurements before and after the operation reported by the patient. We considered this work to be beyond the scope of the first version of the SURPAS tool, but we plan to explore this further in the future.

We have compared the SURPAS models to the American College of Surgeons Surgical Risk Calculator, finding good correlation for overall morbidity [12]. Although we performed an internal validation of the new SURPAS predictive models in this paper and found that they validated well, the SURPAS models also need to be validated externally in future prospective studies.

We view the development, implementation, and dissemination of the SURPAS tool at UCHealth as a long-term project. In addition to exploring patient reported outcomes, future research involving SURPAS will need to address issues such as how to define patients at “high risk” for adverse outcomes, and identifying and testing processes of care that might mitigate their risk and consequently prevent postoperative complications in patients.

## Conclusion

Based upon focus groups of patients, surgeons, and administrators we made refinements to SURPAS. These were successful in improving the accuracy of the models, while reducing manual entry to five of the eight variables. Adding a predictor variable to indicate a complex operation with multiple current procedure codes did not improve the accuracy of the models, therefore SURPAS will use of the primary procedure (CPT code with the greatest wRVU). We developed graphical displays of risk for providers and patients – a take-home handout for patients and an automated documentation of risk and the discussion in the electronic health record. These improvements should facilitate easier use and implementation of SURPAS. Future prospective external validation studies of the SURPAS models are needed.

## Additional file

Additional file 1: **Table S1.** Patient Characteristics and Adverse Outcome Rates for Study Cohort. **Figure S1.** Strengthening the Reporting of Observation studies in Epidemiology (STROBE) diagram. (DOCX 31 kb)

## Data Availability

The datasets used and analyzed during the current study are the ACS NSQIP PUF. These data are the property of the American College of Surgeons, and are freely available to faculty and staff at institutions participating in the ACS NSQIP.

## Abbreviations

ACS NSQIP PUF: American College of Surgeons’ National Surgical Quality Improvement Program Participant Use File
AHRQ: Agency for Healthcare Research and Quality
ASA class: American Society of Anesthesiology physical status classification
COMIRB: Colorado Multiple Institutional Review Board
CPT: Current Procedural Terminology
EHR: Electronic health record
FAQs: Frequently asked questions
FHS: Functional health status prior to surgery
SURPAS: Surgical Risk Preoperative Assessment System
UTI: Urinary tract infection
wRVU: Work Relative Value Unit
