# Supplementary material for: Refining the predictive variables in the “Surgical Risk Preoperative Assessment System” (SURPAS): a descriptive analysis
Source: Patient Saf Surg. 2019 Aug 20;13:28. doi: 10.1186/s13037-019-0208-2 (PMC6702720; doi:10.1186/s13037-019-0208-2)
Supplement: Supplementary file 1 — Table S1. Patient Characteristics and Adverse Outcome Rates for Study Cohort. Figure S1. Strengthening the Reporting of Observation studies in Epidemiology (STROBE) diagram. (DOCX 31 kb) [file 13037_2019_208_MOESM1_ESM.docx]

Table S1. Patient Characteristics and Adverse Outcome Rates for Study Cohort

| Characteristics | | Study Cohort |
| --- | --- | --- |
|  |  | (n=4,543,435)† |
|  | |  |
| **Demographics** | |  |
| Gender | |  |
|  | Female | 2,600,462 (57.2) |
|  | Male | 1,939,003 (42.7) |
|  | Unknown | 3,970 (0.1) |
| Age (years), mean (SD)‡ | | 56.2 (16.9) |
| Race/Ethnicity | |  |
|  | White, not of Hispanic origin | 3,141,676 (69.1) |
|  | Hispanic Origin | 263,295 (5.8) |
|  | Black, not of Hispanic origin | 441,432 (9.7) |
|  | Asian or Pacific Islander | 132,210 (2.9) |
|  | American Indian or Alaska Native | 29,663 (0.7) |
|  | Unknown | 535,159 (11.8) |
|  | |  |
| **Patient Risk Factors** | |  |
| American Society of Anesthesiologist class‡ | |  |
|  | I (A normal healthy patient) | 428,279 (9.4) |
|  | II (A patient with mild systemic disease) | 2,074,009 (45.6) |
|  | III (A patient with severe systemic disease) | 1,766,502 (38.9) |
|  | IV (A patient with severe systemic disease that is a constant threat) | 265,090 (5.8) |
|  | V (A moribund patient who’s not expected to survive) | 9,555 (0.2) |
| Systemic sepsis (within 48 hours)‡ | |  |
|  | None | 4,246,431 (93.5) |
|  | Other | 12,125 (0.3) |
|  | systemic inflammatory response syndrome (SIRS) | 166,439 (3.7) |
|  | Sepsis | 93,612 (2.1) |
|  | Septic Shock | 24,828 (0.6) |
| Functional health status prior to surgery‡ | |  |
|  | Independent | 4,363,933 (96.0) |
|  | Partially Dependent | 138,629 (3.12) |
|  | Totally Dependent | 40,873 (0.9) |
|  | |  |
| **Operative Variables** | |  |
| Work relative value unit, mean (SD)‡ | | 16.2 (10.0) |
| Inpatient/Outpatient operation‡ | |  |
|  | Inpatient | 2,823,793 (62.2) |
|  | Outpatient | 1,719,642 (37.8) |
| Primary surgeon specialty‡ | |  |
|  | Orthopedic surgery | 705,729 (15.5) |
|  | General surgery | 2,549,612 (56.1) |
|  | Gynecology surgery | 270,306 (10.0) |
|  | Urologic surgery | 201,046 (4.4) |
|  | Neurosurgery | 177,236 (3.9) |
|  | Otolaryngology | 104,634 (2.3) |
|  | Thoracic surgery | 48,243 (1.1) |
|  | Plastics surgery | 103,438 (2.3) |
|  | Vascular surgery | 383,191 (8.4) |
| Emergency operation‡ | |  |
|  | No | 4,065,853 (89.5) |
|  | Yes | 477,582 (10.5) |
|  | |  |
| **Outcome Variables** | |  |
| 30-day mortality | |  |
|  | No | 4,488,751 (98.8) |
|  | Yes | 54,684 (1.2) |
| One or more complications | |  |
|  | No | 3,9778,605 (87.6) |
|  | Yes | 564,830 (12.4) |
| Unplanned readmission§ | |  |
|  | No | 2,623,688 (94.7) |
|  | Yes | 146,485 (5.3) |
| Infection complications | |  |
|  | No | 4,316,434 (95.0) |
|  | Yes | 227,001 (5.0) |
| Transfusion complications | |  |
|  | No | 4,319,588 (95.1) |
|  | Yes | 443,847 (4.9) |
| Pulmonary complications | |  |
|  | No | 4,412,968 (97.1) |
|  | Yes | 130,467 (2.9) |
| Urinary tract infection | |  |
|  | No | 4,475,799 (98.5) |
|  | Yes | 67,636 (1.5) |
| Venous thromboembolism (VTE) complications | |  |
|  | No | 4,503,679 (99.1) |
|  | Yes | 39,756 (0.9) |
| Cardiac complication | |  |
|  | No | 4,514,244 (99.4) |
|  | Yes | 29,191 (0.6) |
| Renal complications | |  |
|  | No | 4,515,578 (99.4) |
|  | Yes | 27,857 (0.6) |
| Neurologic complication | |  |
|  | No | 4,534,015 (99.8) |
|  | Yes | 9,420 (0.2) |
| *Abbreviations: IQR, interquartile range; SD, Standard deviation. | | |
| †All values are frequency and percent unless otherwise specified. | | |
| ‡Member of the eight variable Surgical Risk Preoperative Assessment System (SURPAS) prediction models for the eight postoperative outcomes. | | |
| §Unplanned readmission sample sizes was N=2,770,173. | | |

Figure S1. Strengthening the Reporting of Observation studies in Epidemiology (STROBE) diagram.

Patients in ACS NSQIP PUF* 2005-2015

N=4,609,299

Not in target surgeon specialty†

N=26,867 (0.6%)

Within target surgeon specialty†

N=4,582,432 (99.4%)

Missing key data

- Functional health status prior to surgery N=21,896 (0.5%)
- ASA class* N=13,812 (0.3%)
- Age N=3,261 (0.1%)
- Emergency operation status N=25 (<0.1%)
- Inpatient/outpatient N=3 (<0.1%)

Study cohort

N=4,543,435 (98.6%)

*Abbreviations: ACS NSQIP PUF, American College of Surgeon’s National Surgical Quality Improvement Program Participate Use File; ASA, American Society of Anesthesiologists.

†Ophthalmology, oral surgery, podiatry, interventional radiology, cardiac surgery, other and unknown were excluded.
